# Non-destructive analysis of *Ganoderma lucidum* composition using hyperspectral imaging and machine learning

**DOI:** 10.3389/fchem.2025.1534216

**Published:** 2025-02-26

**Authors:** Jing Ran, Hui Xu, Zhilong Wang, Wei Zhang, Xueyuan Bai

**Affiliations:** Northeast Asia Institute of Traditional Chinese Medicine, Changchun University of Chinese Medicine, Changchun, Jilin, China

**Keywords:** polysaccharide, ergosterol, hyperspectral imaging, machine learning model, medicinal fungus

## Abstract

**Background:**

*Ganoderma lucidum* is a widely used medicinal fungus whose quality is influenced by various factors, making traditional chemical detection methods complex and economically challenging. This study addresses the need for fast, noninvasive testing methods by combining hyperspectral imaging with machine learning to predict polysaccharide and ergosterol levels in *Ganoderma lucidum* cap and powder.

**Methods:**

Hyperspectral images in the visible near-infrared (385–1009 nm) and short-wave infrared (899–1695 nm) ranges were collected, with ergosterol measured by high-performance liquid chromatography and polysaccharides assessed via the phenol-sulfuric acid method. Three machine learning models—a feedforward neural network, an extreme learning machine, and a decision tree—were tested.

**Results:**

Notably, the extreme learning machine model, optimized by a genetic algorithm with voting, provided superior predictions, achieving *R* ^2^ values of 0.96 and 0.97 for polysaccharides and ergosterol, respectively.

**Conclusion:**

This integration of hyperspectral imaging and machine learning offers a novel, nondestructive approach to assessing *Ganoderma lucidum* quality.

## 1 Introduction

*Ganoderma lucidum*, a member of the Ganodermataceae family, is classified as a white-rot fungi (Wang T. T. et al., 2024). This species primarily grows in tropical, subtropical, and temperate climates. As one of the leading producers, China has developed a large-scale *Ganoderma lucidum* planting industry. This fungus is rich in specific bioactive components, including polysaccharides, triterpenes, proteins, and sterols (Cör et al., 2022). *Ganoderma lucidum*, known for its remarkable antioxidant, antibacterial, tumor-inhibiting, and anti-inflammatory effects, holds a significant position in the healthcare and nutrition market. This is attributed to its three primary functions: nourishment, treatment, and tonification. As awareness of the quality of *Ganoderma lucidum* increases, it becomes evident that its quality is significantly affected by many factors, including producing area, cultivation environment, harvest conditions, and so on. Researchers have developed various quality control techniques for *Ganoderma lucidum,* including UV-Vis spectrophotometry (Jiang et al., 2018) and nuclear magnetic resonance (Wang M. et al., 2024) spectroscopy for analyzing polysaccharides, high-performance liquid chromatography (HPLC) for assessing triterpenoid acids (Zeng et al., 2023) and ergosterol (Liu et al., 2011), HPLC-ELSD lipid profiling (Xia et al., 2023), and high-performance capillary electrophoresis for nuclear glycoside content analysis (Dai et al., 2002). Although these methods can be employed to determine the chemical composition of *Ganoderma lucidum*, traditional detection techniques have limitations, including complexity, time consumption, destructiveness, and sensitivity to standard experimental procedures.

Advancements in spectral technology have significantly improved non-destructive testing methods. Consequently, researchers have quantitatively analyzed polysaccharides and triterpene compounds in *Ganoderma lucidum* (Zhang and Huang, 2021; Zhang, 2020) alongside rapid identification of *Ganoderma lucidum* varieties (Yang et al., 2017) through near-infrared fingerprint technology. Near-infrared spectroscopy works by measuring samples that absorb near-infrared light, which provides insight into their chemical composition and physical properties. However, this technique typically offers lower spatial resolution and is limited to spectroscopic information. Hyperspectral imaging technology (HSI)—a novel analytical method—integrates traditional imaging with spectroscopy to simultaneously capture spatial and spectral information of samples. This technology offers several advantages: it is non-contact, non-destructive, fast, and capable of providing a large amount of information. It holds significant potential for applications across agriculture, food, medicine, and other fields. Machine learning, an important branch of artificial intelligence, automatically extracts features from large datasets and learns rules by creating predictive models to accurately predict or classify unknown samples. Xu et al. employed principal component analysis (PCA) to extract feature bands and utilized a support vector machine for modeling moldy walnuts (Xu et al., 2022). Xiao et al. developed an SVM model using feature wavelengths extracted via PCA and convolutional neural networks, achieving results comparable to or better than those of the full-wavelength model (Xiao et al., 2020). Li et al. extracted 13 characteristic wavelengths using a stacked autoencoder and achieved optimal predictive performance in a GA-ELM model, with an *R* ^2^ value of 0.97 (Li et al., 2023a). This study achieved an overall classification accuracy of 93%. Furthermore, Li et al. successfully used the SNV-SPA-LS-SVM algorithm to detect protein content in mulberry leaves. This method effectively assessed leaf quality, yielding an RPD value of 3.83 and an R^2^for protein detection (Li et al., 2023b).

The researchers captured hyperspectral images of wheat flour in the range of 968–2576 nm and developed models using partial least squares discriminant analysis and SVM based on characteristic wavelengths. The results indicate that the nonlinear discriminant model outperformed the linear model in classifying the wheat flour grades. Furthermore, the MSC-UVE-CARS-PSO-SVM model provides superior prediction performance, achieving 100% accuracy in the calibration and validation sets (Zhang et al., 2023). Alfaro-Mejía et al. propose an unsupervised deep learning model for endmember extraction and fractional abundance map estimation from the HSI (Alfaro-Mejía et al., 2023). Other scholars have used a fast and compact Hybrid CNN to process HSI data for bloodstain identification and classification (Butt et al., 2022). Hou et al. proposed a hyperspectral imagery classification model based on self-supervised contrastive learning (SSCL),where conventional spectral-spatial features and deep models are combined to improve the classification accuracy (Hou et al., 2021). Advancements in HSI and machine learning algorithms are further improving their application in the quality detection of *Ganoderma lucidum*. For example, Pan et al. and other researchers developed a prediction model by collecting hyperspectral images of *Ganoderma lucidum* spore powder samples with varying wall-breaking rates (Pan et al., 2024). The model incorporated spectral preprocessing and characteristic band extraction. The combination of Savitzky-Golay smoothing and competitive adaptive reweighted feature band selection together with partial least squares yielded the best prediction performance. This technique successfully enabled a rapid, non-destructive testing approach for determining the wall-breaking rate of *Ganoderma lucidum* spore powder. Additionally, other researchers have employed visible-near infrared HSI to pre-detect polysaccharide content in *Ganoderma lucidum* fruiting bodies. *Ganoderma lucidum* polysaccharide content has been accurately predicted by collecting hyperspectral images, conducting spectral preprocessing, extracting characteristic bands, and utilizing partial least squares modeling (Liu Y. et al., 2021). The concentration of 15 inorganic elements, including lead, in the entire fruiting body of *Ganoderma lucidum* was higher than those in the caps (Liu et al., 2023). Furthermore, while the caps contain higher concentrations of ganoderic acid A (Huang et al., 2017) and ganoderic acid B (Shi et al., 2013), infrared spectroscopy indicates that the levels of amino acids, peptides, and proteins in *Ganoderma lucidum* were higher than those in the caps (Huang et al., 2015).

In a previous hyperspectral study of *Ganoderma lucidum* polysaccharides, only the cap was collected. However, differences in the distribution of chemical compositions in *Ganoderma lucidum* may result in inaccurate model predictions. Therefore, hyperspectral images of *Ganoderma lucidum* caps and powder were collected, and the hyperspectral data were preprocessed using genetic algorithms and principal component analysis, and three kinds of machine learning models, BPNN, ELM, and DT, of the original spectral data and the preprocessed spectral data, were established for the prediction of the chemical content of the samples of different morphologies of *Ganoderma lucidum*, and the best prediction models were screened out by comparing different algorithms with each other. It aims to provide a new method for non-destructive testing of *Ganoderma lucidum* quality.

## 2 Materials and methods

### 2.1 Sample preparation and hyperspectral image acquisition

In this study, 32 fruiting bodies of *Ganoderma lucidum* were collected from 13 production areas. Table 1 provides detailed information. Upon collection, the *Ganoderma lucidum* cap was immediately crushed into powder, which was pressed onto A4 paper to coin-sized shapes for consistent hyperspectral image acquisition under the same conditions. During the collection, the indoor temperature was kept at approximately 25°C. Each sample was photographed three times, and the average spectrum was calculated from the original spectral data.

**TABLE 1:** Samples of *Ganoderma lucidum*.

Origins	Numbers	Identification
Jilin Fusong	4	A1
Jiaohe, Jilin	3	A2
Huadian, Jilin	1	A3
Jilin City, Jilin Province	3	A4
Tonghua, Jilin	3	A5
Anqing, Anhui	1	B2
Bozhou, Anhui	3	B3
Tai’an, Shandong	3	C1
Liaocheng, Shandong	1	C2
Jixi, Heilongjiang	3	D
Longquan, Zhejiang	1	E
Baise, Guangxi	3	F
Linzhi, Tibet	3	G

**FIGURE 1:**
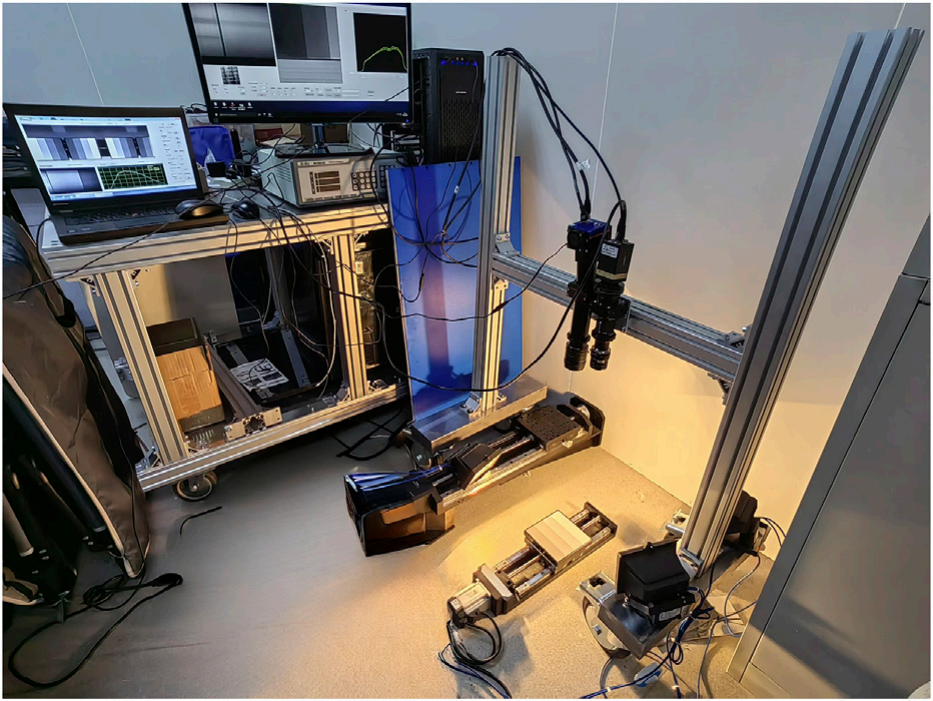
Hyperspectral imaging system.

The HSI system used for image acquisition adopts a dual-light source set-up, consisting of a grating splitter, charge-coupled device, an IMPERX 1920 × 1080 visible near-infrared camera (lens: Kowa 35 mm focal length), Guohui 640 × 512 short-wave infrared camera (lens: AZURE 50 mm focal length), halogen light sources, electric platform, and computer. The experimental equipment was provided by the Changchun Institute of Optics and Precision Machinery, Chinese Academy of Sciences (Figure 1).

Calibration images were collected (Reddy et al., 2023) before acquiring the hyperspectral images to correct for any variations in light source intensity and dark current, ensuring accurate spectral data (Dai et al., 2023). The calibration formula is in the following Equation 1.

$$R=\frac{I_{raw}-I_{dark}}{I_{white}-I_{dark}}$$ (1)

where R is the calibrated reflectance image, I_raw_ is the original reflectance image, I_dark_ represents the blackboard reference image, and I_white_ is the whiteboard reference image.

**FIGURE 2:**
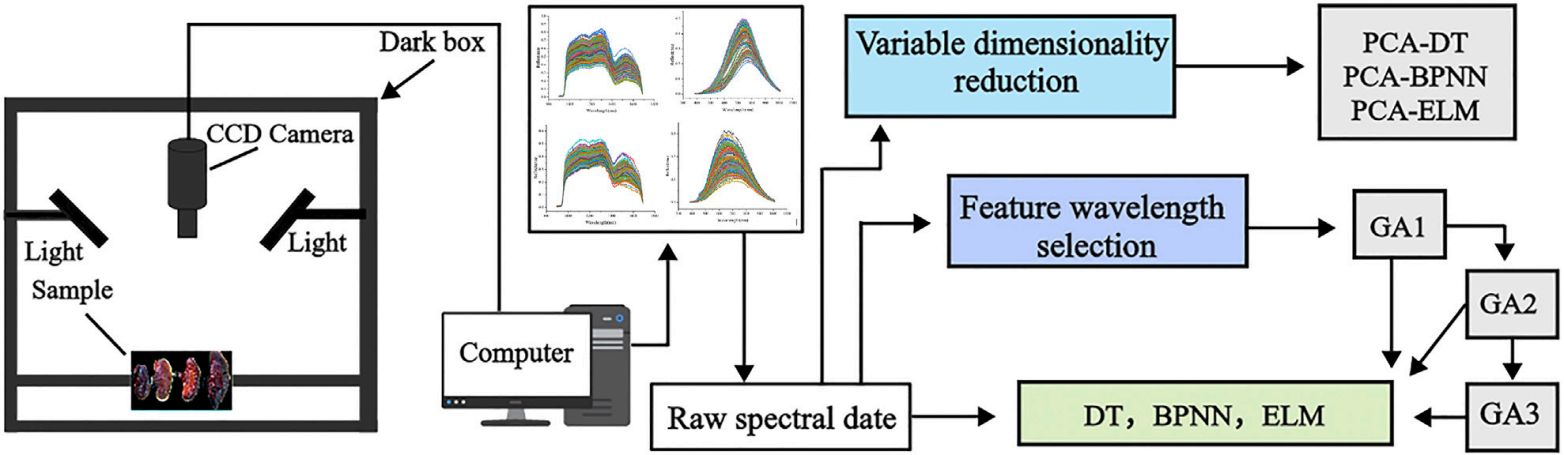
Schematic diagram of *Ganoderma lucidum* sample program analysis.

Figure 2 Provides a schematic overview of the analysis process, including the acquisition of spectral data and model development.

### 2.2 Region of interest extraction and sample set division

The region of interest was manually delineated using ENVI5.3 software, selecting three pixels and calculating their average values as the spectral reflectance for each sample. Overall, 128 regions of interest were extracted from 32 *Ganoderma lucidum* cap (GLC) and powder (GLP), which were randomly divided into training and prediction sets at a ratio of 115:13 for subsequent modeling.

### 2.3 Measurement of chemical composition

#### 2.3.1 Polysaccharides

To measure polysaccharide content, 1.0 g of the fruiting body powder of *Ganoderma lucidum* collected from the hyperspectral images was weighed, and 40 mL of pure water was added. The mixture was then boiled for 2 h, fixed volume to 50 mL, and filtered. The filtrate (5 mL) was added to 25 mL of anhydrous ethanol, followed by overnight precipitation. The mixture was centrifuged at 5000 rpm for 25 min. The resulting supernatant was discarded, and the residue was dissolved in 25 mL of water. The solution (1 mL) was thoroughly mixed with water to 2 mL, and the absorbance was determined according to the phenol sulfate method. The determination was repeated three times, and the average value was calculated.

#### 2.3.2 Ergosterol

A 1.0 g sample of the powder collected from the HSI was dissolved in 20 mL of methanol, thoroughly mixed, and sonicated for 1 h. Following filtration, the ergosterol content in *Ganoderma lucidum* was determined using HPLC. The HPLC conditions were as follows: an Agilent Eclipse Plus C18 column (4.6 mm × 250 mm,5 μm), flow rate of 1.0 mL/min, detection wavelength of 282 nm, injection volume of 10 μL, column temperature of 30°C, and methanol as mobile phase.

### 2.4 Hyperspectral data processing methods

Hyperspectral data is high-dimensional and nonlinear, often containing large volumes of information, noise, redundancy, and collinearity, all of which complicate analysis (Chen et al., 2024; Sun et al., 2024). Using full-spectrum modeling not only increases the complexity of model training but also causes overfitting. Therefore, reducing variables and selecting relevant wavelengths are essential for enhancing classification accuracy and simplifying computations.

#### 2.4.1 Variable dimensionality reduction

Dimensionality reduction is an essential preprocessing step in data mining with broad applications, particularly for spectral data. PCA is a commonly used unsupervised learning method.

PCA uses an orthogonal transformation to convert observed data represented by linearly correlated variables into a smaller set of linearly independent variables, known as principal components. Principal components with high interpretive value retain most of the critical information from hyperspectral images (Xiao et al., 2020; Ye et al., 2022).

#### 2.4.2 Characteristic wavelength selection

A GA is an optimization method inspired by the principles of natural selection and Darwinian evolution theory. Following the “survival of the fittest” principle, GA uses the “network” to find the global optimal solution by simulating the biological evolution process (Zhang et al., 2024). Using “minimum error” as the evolutionary criterion, the optimal solution is searched in the global scope (Wang et al., 2021). Compared with the inverse gradient descent method, GA can search multiple solutions within the solution space simultaneously without requiring differential operation. This makes GA highly robust and widely applicable (Wang et al., 2023). We propose two methods for selecting feature wavelengths using GA. The first is iterative feature selection, which reduces the number of wavelengths over multiple iterations, refining the quality of the selected wavelengths. The second approach uses voting-based feature selection, where multiple runs are performed, and the wavelengths selected most frequently are designated as the characteristic wavelength. The roulette wheel selection strategy was used to select the most fit individual. Additionally, single-point crossover was implemented to improve the search process. The variation operator uses the fundamental bitwise variation and sets the variation probability to 0.05. This setting helped prevent the algorithm from getting stuck in a local optimum. It also enhanced its ability to perform global searches. These genetic operators collaborate to help the algorithm discover a better combination of features in the feature selection process, which ultimately improves the final model’s performance.

### 2.5 Model construction and evaluation

#### 2.5.1 BP neural network

**FIGURE 3:**
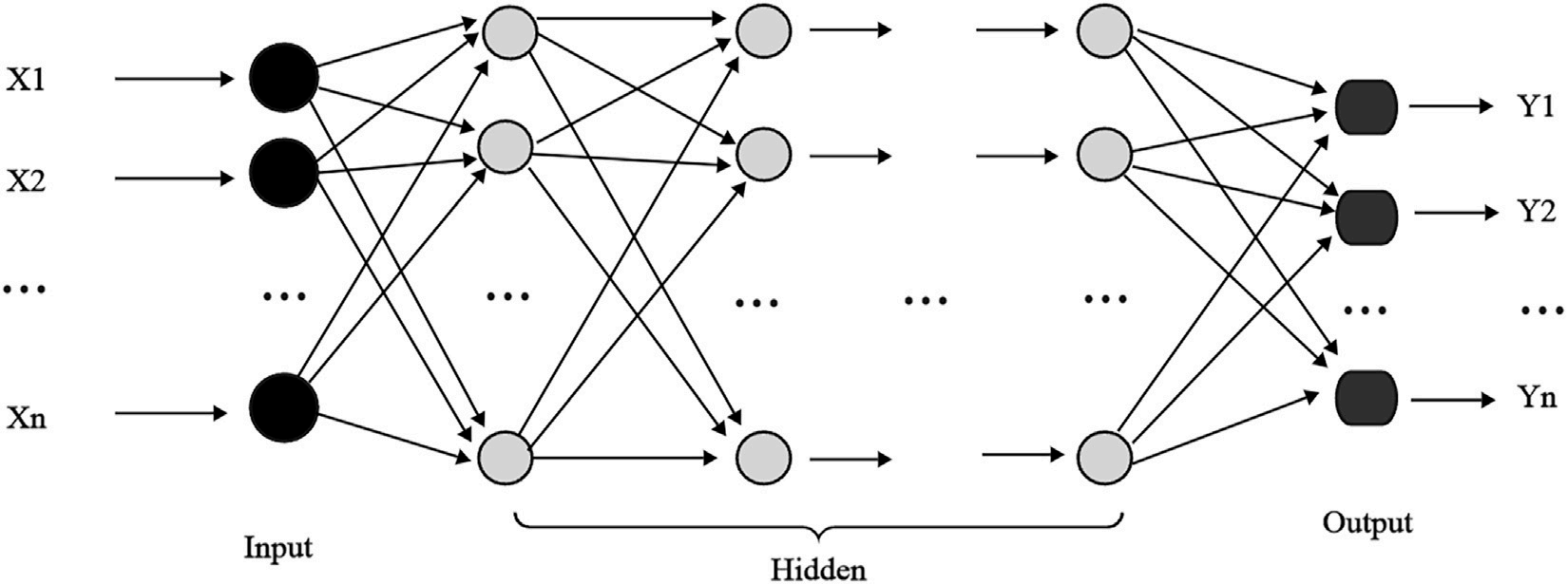
Topology diagram of backpropagation neural network.

A back propagation (BP) neural network is a multilayer feed forward network and one of the most widely used neural network architectures (Figure 3). Each BP neural network (Yu and Sun, 2024) consists of an input, hidden, and output layer, where there can be one or more hidden layers (He and Zhou, 2022). The core concept of a BP neural network is to adjust the connection weight of the network through the backpropagation error, thus minimizing the output error. However, in complex networks, training can often become trapped in local minimum (Yuan et al., 2009). Additionally, the performance of BP neural networks largely depends on the settings of hyperparameters, such as the learning rate and number of hidden layers and neurons. If the learning rate is too high, training may become unstable, while too low may cause slow or stalled training. Increasing hidden layers or nodes can reduce error and improve model generalization, simultaneously increasing neural network complexity, extending learning time, and may eventually cause overfitting. Overfitting makes the model prone to getting trapped in a local minimum, ultimately degrading its e performance. Conversely, reducing the number of hidden layers may prevent the network from achieving sufficient accuracy or cause underfitting. Therefore, balancing the number of nodes is essential for optimizing model performance, minimizing overfitting, and achieving a robust fit (Liu et al., 2016). In this study, we configure the BPNN with the following parameters: the hidden layer contains 100 neurons, the maximum number of iterations is set to 1000, the initial learning rate is 0.01, the activation function is the tanh, and the solver is the lbfgs algorithm.

#### 2.5.2 Decision tree

**FIGURE 4:**
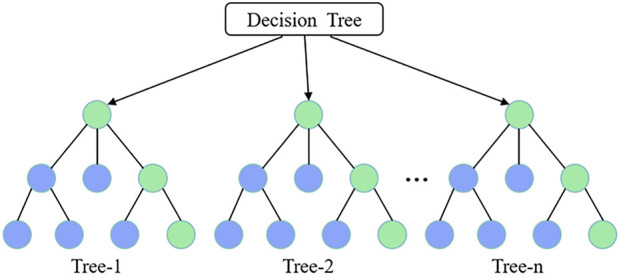
Topology diagram of the decision tree.

A decision tree (DT) is a machine-learning model that uses a hierarchical tree structure composed of a root node, several internal nodes, and leaf nodes to represent decision rules and classification outcomes. The root node serves as the starting point of the tree, each internal node represents a feature attribute or judgment condition (Xu et al., 2022), and the leaf node represents the final prediction result (Figure 4). The decision tree recursively partitions the dataset until the stopping condition is met, thus forming a prediction model (Li M. Y., 2024). Model stability generally increases with a lower learning rate. Additionally, the complexity of the model is influenced by the tree depth and number of splits: as complexity increases, more decision trees are required, which increases training time and storage requirements (Zhang et al., 2024).

#### 2.5.3 Extreme learning machine

**FIGURE 5:**
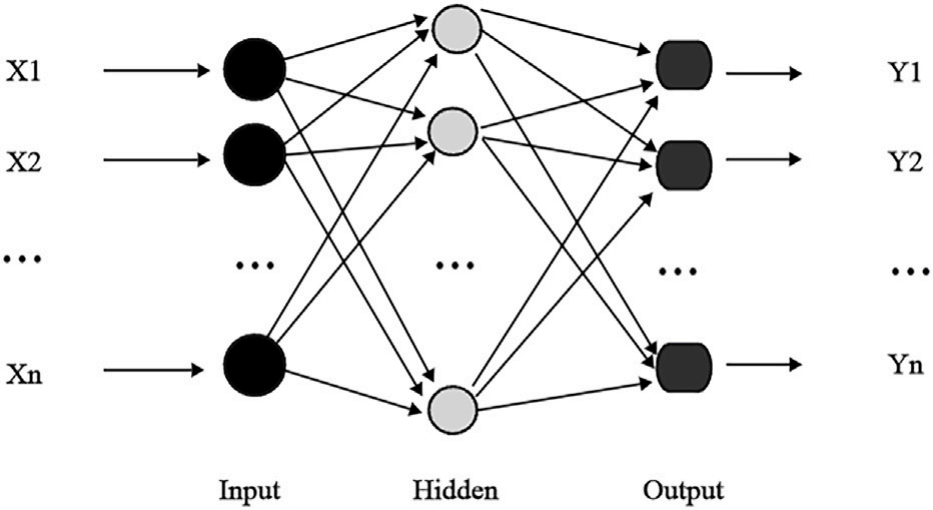
Topology diagram of the extreme learning machine.

Extreme learning machine (ELM) is a single-layer feedforward neural network consisting of an input, a hidden, and an output layer (Figure 5), known for its strong performance in classification and regression (Jia et al., 2024; Huang et al., 2006; Liu et al., 2022; Feng et al., 2024). The principle of the ELM is to map the input data into a high-dimensional feature space to establish an optimal hyperplane that best represents the relationship between inputs and outputs (Li P. X., 2024). Unlike traditional feedforward neural networks, ELM reduces complexity by avoiding iterative learning and parameter tuning, transforming the training into a simple matrix inversion problem. Hidden layer weights and thresholds are assigned randomly, while output layer weights are directly calculated, bypassing gradient descent. Therefore, ELM significantly reduces training time and offers high learning efficiency, accuracy, and ease of parameter adjustment (Chen and Wang, 2019; Lu et al., 2024; Liu T. et al., 2021).

### 2.6 Model evaluation

The training set determination coefficient (R_T_^2^), verification set determination coefficient (R_V_^2^), and mean square error (MSEP) are utilized as indicators to determine the model performances. All the models ran 10 times for averaging to ensure the robustness of the model evaluation. The closer the determination coefficient (*R* ^2^) is to 1, the better the fitting effect of the model (Li et al., 2023c). Among them, *R* ^2^ > 0.90 indicates excellent prediction ability of the model, 0.81 < *R* ^2^ < 0.90 suggests good prediction performance of the model, and 0.60 < *R* ^2^ < 0.80 shows that the model is general but still useable for prediction. The MSEP is employed to determine the deviation between the predicted and real values. A larger *R* ^2^ and a smaller MSEP indicates higher accuracy and better stability of the model (Cui et al., 2017).

## 3 Results

### 3.1 Chemical composition content

**FIGURE 6:**
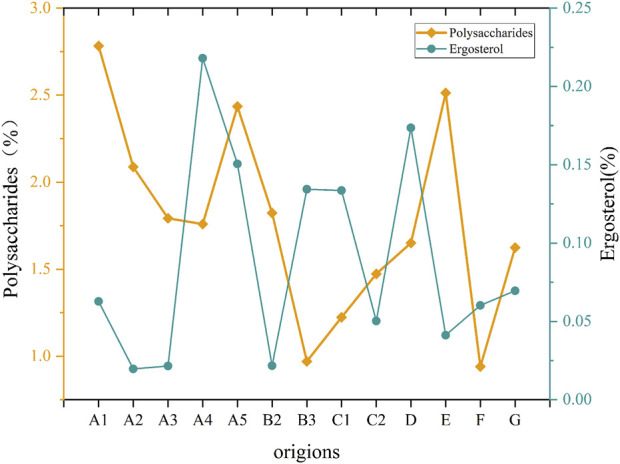
Diagram of chemical composition content in *Ganoderma lucidum* samples.

By mapping the average content of *Ganoderma lucidum* polysaccharides and ergosterol (Figure 6), the polysaccharide content in Bozhou, Anhui, and Baise, Guangxi, was <1.0%, which was the lowest among all the producing areas. In contrast, ergosterol content was relatively high in Longquan, Zhejiang, and Fusong, Jilin Province, with the highest levels found in Jilin City, Jilin Province. These findings suggest significant variation in *Ganoderma lucidum* contents across different areas; however, traditional separation and detection methods were time-consuming and complex. Furthermore, the performance of each component differs across production areas, complicating the evaluation of *Ganoderma lucidum* quantity based solely on a single component. Therefore, using hyperspectral technology to predict the content and assess the quality of *Ganoderma lucidum* may be highly valuable.

### 3.2 Spectral characteristics

**FIGURE 7:**
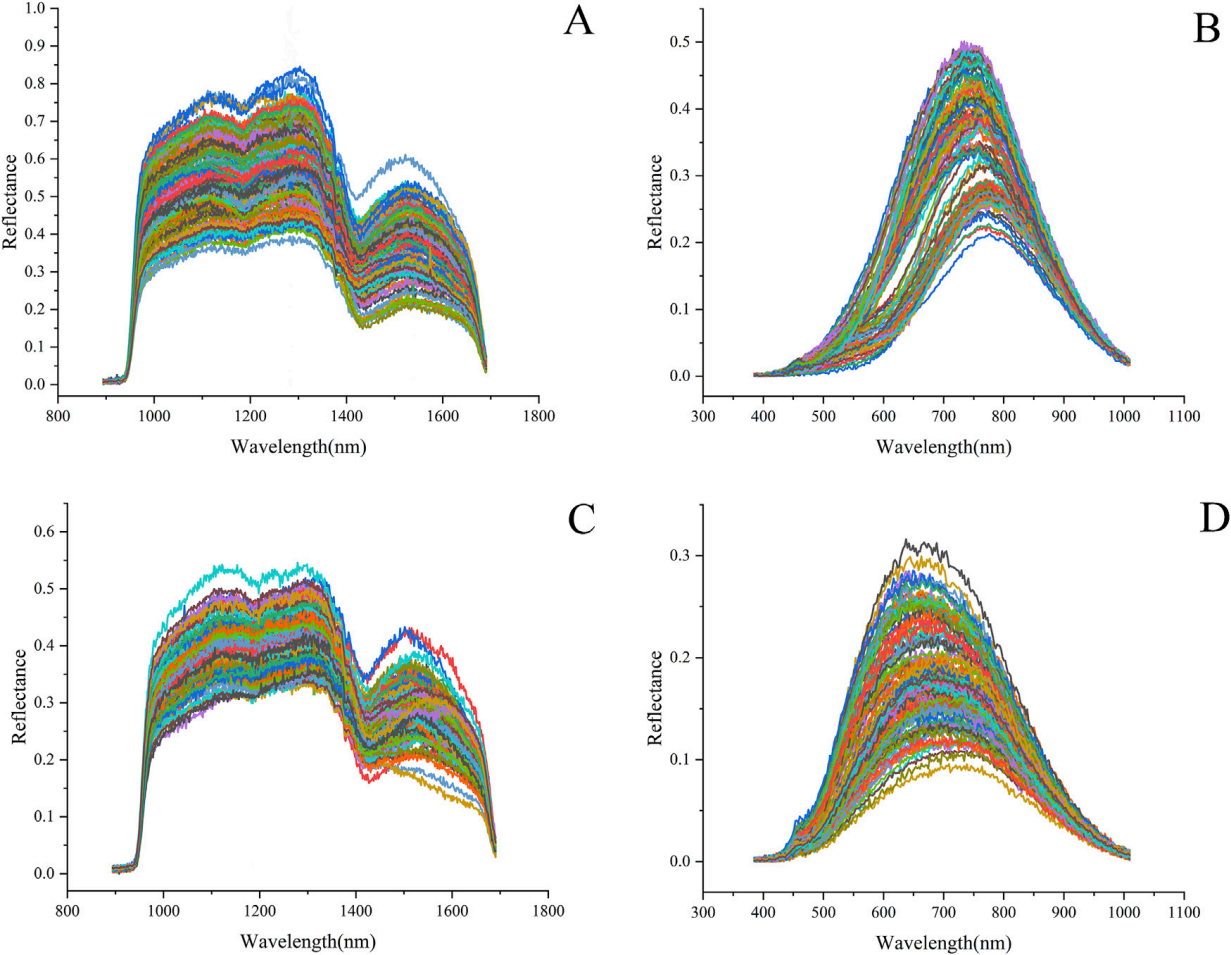
Spectral reflectance map of *Ganoderma lucidum* samples: **(A)** GLC spectrum in the range of SWIR and **(B)** VNIR. **(C)** GLP spectrum in the range of SWIR and **(D)** VNIR.

The spectral reflectance of the GLC and GLP showed similar trends in different wavelength bands, but their reflectance values exhibited significant differences (Figure 7). The average reflectance of the GLC was higher than that of the GLP. In the range of the VNIR band, the reflectance map of the GLC appeared delaminated, owing to structural variation, such as surface roughness and texture. After crushing, the sample exhibited high uniformity, causing this delamination effect to disappear and shifting the position of the absorption peak accordingly. Absorption peaks were observed near 1100, 1300, and 1520 nm in the SWIR band; the peak at 1100 nm may be related to the second overtone of N-H stretching, while that at 1300 nm may be attributed to the second overtone of C-H stretching. A local minimum is observed near 1200 and1430 nm; the trough at 1200 nm may correspond to the ergosterol absorption band in *Ganoderma lucidum* (Delwiche et al., 2019), and those at 1430 and 1520 nm may be related to the first overtone of O-H stretching.

### 3.3 Modeling and analysis

Through the modeling of polysaccharide and ergosterol contents in the GLC and GLP (Table 2), the training accuracy of all models was >0.90, demonstrating strong performance. However, model effects varied significantly across different band ranges. In the SWIR band range, the BPNN and ELM models performed better than those of the other models. The prediction of DT model results showed that the GLC contained significantly higher levels of polysaccharides and ergosterol compared to that of the GLP. The prediction accuracy for the GLP was below 0.43, which was insufficient for effective prediction. In the BP model, the accuracy for predicting polysaccharides in the GLP slightly exceeded that of the GLC, while the model achieved the highest accuracy for predicting ergosterol, with an *R* ^2^ value of 0.94.

**TABLE 2:** Predictive analysis based on original spectral modeling.

		SWIR						VNIR					
		GLC			GLP			GLC			GLP		
		R_P_^2^	MSEP	R_T_^2^	R_P_^2^	MSEP	R_T_^2^	R_P_^2^	MSEP	R_T_^2^	R_P_^2^	MSEP	R_T_^2^
polysaccharides	DT	0.79	0.16344	0.93	0.43	0.54344	0.86	0.70	0.39496	0.92	0.72	0.13792	0.94
	BP	0.90	0.06663	0.96	0.91	0.04238	0.98	0.92	0.11338	0.95	0.96	0.03277	0.97
	ELM	0.94	0.08107	0.90	0.92	0.08555	0.92	0.92	0.02883	0.93	0.91	0.06044	0.93
ergosterol	DT	0.77	0.00090	0.96	0.28	0.00116	0.92	0.92	0.00024	0.96	0.86	0.00047	0.98
	BP	0.91	0.00045	0.94	0.94	0.00041	0.96	0.92	0.00024	0.95	0.90	0.00030	0.99
	ELM	0.91	0.00059	0.93	0.91	0.00029	0.93	0.96	0.00033	0.96	0.92	0.00041	0.92

In the VNIR band range, the BP model showed the optimal prediction effect for polysaccharides in the GLP, achieving an R^2^of 0.96. In contrast, the ELM model predicted ergosterol in the GLC with an *R* ^2^ of 0.96. The performance of the DT model for GLP significantly exceeded that of the SWIR band, particularly in predicting ergosterol.

### 3.4 Characteristic wavelength selection

#### 3.4.1 Result of characteristic wavelength selection

**FIGURE 8:**
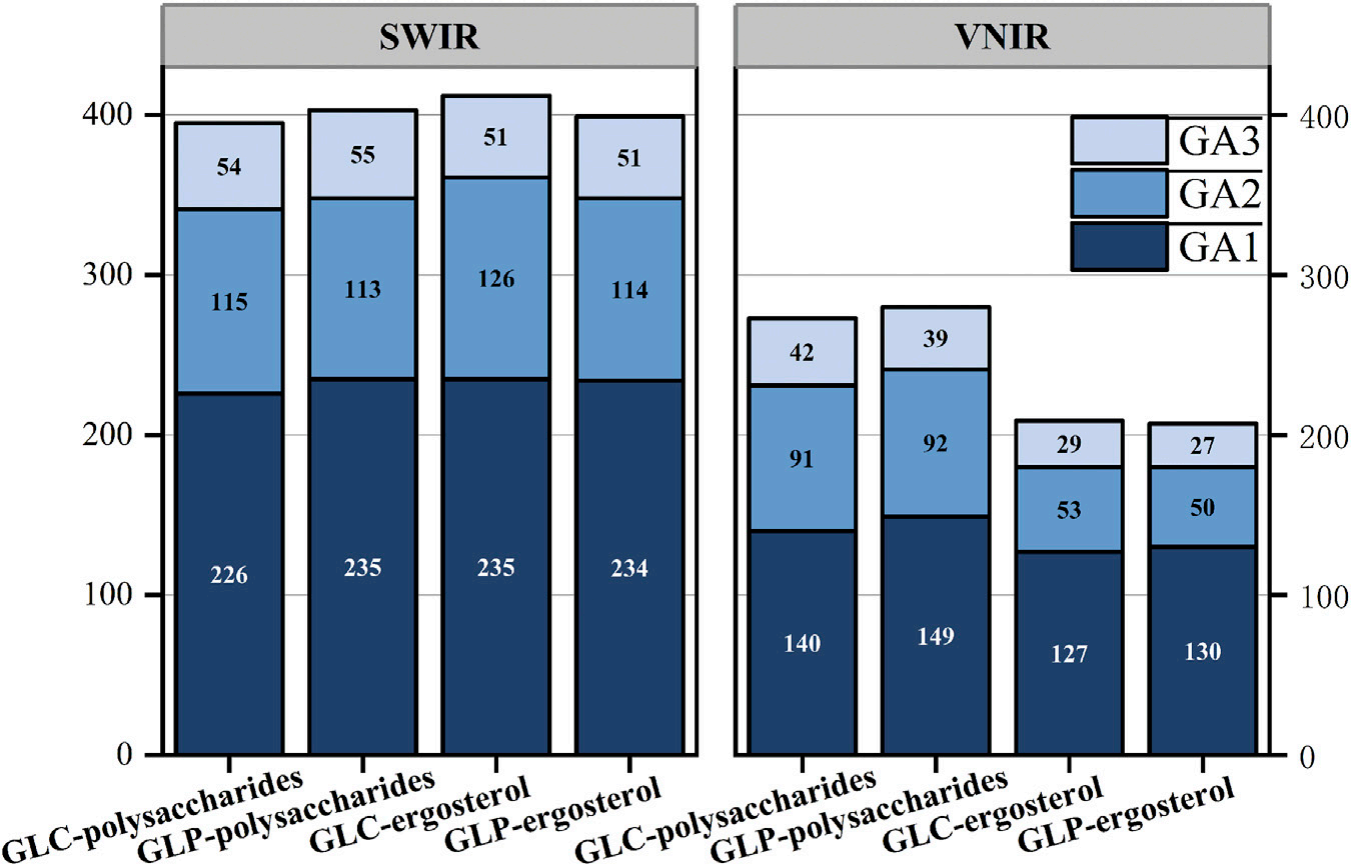
Number of characteristic wavelengths.

Two methods for feature-wavelength selection were proposed in this study using the GA. The first method, iterative feature selection, is conducted thrice to obtain different sets of feature wavelengths (Figure 8). The second method is voting feature selection. The second technique, voting feature selection, involves selecting 50 results with a determination coefficient >0.7. The wavelength that appears most frequently is selected as the feature wavelength, matching the count obtained from the iterative feature selection. Polysaccharides select more characteristic wavelengths than ergosterols in the VNIR band. This may be because polysaccharides are polymeric compounds composed of multiple monosaccharide molecules connected by glycosidic bonds that contain numerous functional groups (e.g., OH). Its chemical structure is complex, with various intramolecular and intermolecular interaction types, such as hydrogen bonds and van der Waals forces, which increase the overlap of the spectra. Consequently, identifying the characteristic wavelengths becomes challenging, and the number of these wavelengths is larger.

#### 3.4.2 Characteristic wavelength modeling results

This study employed two GA to extract the characteristic wavelengths and established three models: BPNN, ELM, and DT. The first was used to predict polysaccharides (Table 3). The training accuracy of all models was >0.81, and the MSEP of the verification set was minimal, indicating good performance. When using two types of GA selection across different wavelength ranges, the prediction accuracy for GLC and GLP surpasses that of the characteristic wavelength voting method. In the SWR range, the prediction accuracy of the three models under voting conditions ranked as follows: ELM > BPNN > DT. The ELM model showed the best prediction performance for GLC and GLP, with *R* ^2^ values of 0.96 and 0.95, respectively. The DT model of the GLC performed worse than the original model under two types of GA conditions. However, the prediction accuracy of iterative feature selection is significantly higher than that of voting, with *R* ^2^ values > 0.61. For GLP, the prediction accuracy of the DT model was better than that of the original model when using the voting GA. Across all three models, the prediction accuracy for GLC was higher than that of GLP. In the VNIR range, the prediction accuracy of the DT model was significantly higher than that of SWIR, increasing from 0.23 to 0.90 by the third iteration of GLC. The prediction accuracies of the two GA methods were significantly higher than those of the original models. The prediction accuracy of the three models was consistent within the SWIR range. The GLC achieved the highest prediction accuracy with an *R* ^2^ value of 0.96 in the ELM model under the voting GA condition. Overall, for GLC, the average *R* ^2^ values were 0.82 for iterative GA selection and 0.90 for voting GA, while for GLP, the values were 0.82 and 0.88, respectively.

**TABLE 3:** Modeling and prediction results of polysaccharides based on different GA characteristic wavelength extraction methods.

			SWIR						VNIR					
			GLC			GLP			GLC			GLP		
			R_P_^2^	MSEP	R_T_^2^	R_P_^2^	MSEP	R_T_^2^	R_P_^2^	MSEP	R_T_^2^	R_P_^2^	MSEP	R_T_^2^
Iteration	GA1	DT	0.43	0.37866	0.86	0.22	0.41046	0.85	0.85	0.12045	0.92	0.72	0.21023	0.93
		BP	0.88	0.08973	0.90	0.90	0.09160	0.94	0.92	0.05908	0.93	0.92	0.13836	0.96
		ELM	0.95	0.05354	0.95	0.95	0.04008	0.97	0.96	0.02238	0.98	0.94	0.13313	0.95
	GA2	DT	0.47	0.36487	0.81	0.5	0.29792	0.82	0.84	0.09545	0.89	0.82	0.08886	0.92
		BP	0.91	0.10084	0.91	0.94	0.03787	0.94	0.92	0.07607	0.93	0.91	0.04212	0.95
		ELM	0.92	0.07141	0.96	0.91	0.05589	0.91	0.96	0.04511	0.97	0.93	0.04819	0.94
	GA3	DT	0.23	0.62163	0.81	0.73	0.12729	0.81	0.9	0.00020	0.9	0.73	0.13908	0.94
		BP	0.84	0.19085	0.88	0.94	0.05518	0.96	0.92	0.06307	0.93	0.9	0.12113	0.92
		ELM	0.94	0.05664	0.94	0.88	0.09554	0.94	0.92	0.14226	0.96	0.85	0.34920	0.92
Vote	GA1	DT	0.62	0.41219	0.87	0.61	0.15264	0.81	0.86	0.05895	0.95	0.86	0.44724	0.83
		BP	0.94	0.04855	0.94	0.92	0.06101	0.95	0.96	0.05150	0.96	0.95	0.03769	0.96
		ELM	0.96	0.02695	0.96	0.95	0.04940	0.95	0.96	0.04319	0.98	0.94	0.07691	0.95
	GA2	DT	0.76	0.08397	0.84	0.69	0.10474	0.82	0.91	0.07629	0.92	0.91	0.03906	0.94
		BP	0.94	0.03086	0.94	0.94	0.05594	0.95	0.94	0.01371	0.94	0.93	0.08137	0.95
		ELM	0.96	0.03483	0.96	0.95	0.03872	0.95	0.96	0.03272	0.97	0.94	0.03446	0.97
	GA3	DT	0.76	0.11890	0.88	0.73	0.17983	0.83	0.91	0.07568	0.91	0.76	0.17893	0.93
		BP	0.95	0.08343	0.93	0.94	0.04940	0.96	0.95	0.03238	0.96	0.9	0.12855	0.88
		ELM	0.96	0.04188	0.97	0.95	0.08341	0.92	0.94	0.06976	0.97	0.89	0.22995	0.92

All models for predicting ergosterol levels (Table 4) achieved a training accuracy >0.93, the MSEP of the verification set was minimal, demonstrating effective training. In the SWIR range, the DT model performs worse than that of the BPNN and ELM models. The iterative GA prediction accuracy for ergosterol in GLP was <0.54, and the accuracy of GA1 was lower than that of the original model, with an *R* ^2^ value of only 0.21. The DT model selected by the three-voting GA outperformed the original model, achieving an *R* ^2^ value > 0.61. In contrast, the ELM model in GA3 exhibited a lower performance compared to that of the iterative model. The prediction effect of the three models of GLC under voting GA was ranked as ELM > BPNN > DT, with the ELM achieving the highest accuracy at 0.95. In the iterative GA selection, the BPNN model performed worse than the original model inGA1andGA3, and only the DT model in GA2 outperformed the original model. In the VNIR range, the DT model developed using GLP demonstrated superior performance than that of the SWIR range. The prediction accuracy of the three models assessed in the voting GA3 was slightly lower than that of the iterative GA3. The predictive performance of the three models utilizing GLC *w*as consistent with the SWIR findings, ranking as follows: ELM > BPNN > DT, with ELM achieving the highest prediction accuracy of 0.97. Overall, the average *R* ^2^ of the iterative GA model based on the characteristic wavelengths of the GLC was 0.87; however, the voting GA model achieved 0.91. The iterative GA model using the characteristic wavelengths of GLP resulted in an *R* ^2^ of 0.82, compared to an *R* ^2^ of 0.87 for the voting GA model. In summary, the predictions for ergosterol and polysaccharides are consistent across different models, with the model employing characteristic wavelengths selected by voting GA being the most effective. Among them, the predictive performance of ELM in the GA1 and GA2 in the VNIR band was the highest, with *R* ^2^ of 0.97.

**TABLE 4:** Modeling and prediction results of ergosterol based on different GA characteristic wavelength extraction methods.

			SWIR						VNIR					
			GLC			GLP			GLC			GLP		
			R_P_^2^	MSEP	R_T_^2^	R_P_^2^	MSEP	R_T_^2^	R_P_^2^	MSEP	R_T_^2^	R_P_^2^	MSEP	R_T_^2^
Iteration	GA1	DT	0.73	0.00084	0.97	0.21	0.00302	0.85	0.87	0.00030	0.92	0.87	0.00051	0.98
		BP	0.86	0.00060	0.94	0.92	0.00047	0.96	0.92	0.00044	0.93	0.81	0.00079	0.98
		ELM	0.94	0.00017	0.95	0.92	0.00039	0.93	0.94	0.00021	0.95	0.92	0.00027	0.96
	GA2	DT	0.79	0.00018	0.93	0.54	0.00183	0.91	0.79	0.00079	0.87	0.94	0.00031	0.95
		BP	0.9	0.00036	0.91	0.94	0.00014	0.95	0.92	0.00026	0.93	0.87	0.00044	0.92
		ELM	0.95	0.00020	0.97	0.93	0.00015	0.95	0.93	0.00036	0.95	0.88	0.00149	0.94
	GA3	DT	0.73	0.00058	0.94	0.46	0.00283	0.86	0.77	0.00077	0.83	0.91	0.00037	0.98
		BP	0.94	0.00033	0.95	0.92	0.00043	0.94	0.91	0.00040	0.91	0.92	0.00058	0.93
		ELM	0.95	0.00048	0.97	0.92	0.00283	0.94	0.9	0.00105	0.94	0.89	0.00186	0.94
Vote	GA1	DT	0.75	0.00056	0.92	0.62	0.00119	0.92	0.89	0.00039	0.92	0.88	0.00045	0.94
		BP	0.94	0.00017	0.94	0.95	0.00013	0.97	0.95	0.00019	0.97	0.93	0.00025	0.98
		ELM	0.95	0.00015	0.96	0.93	0.00020	0.94	0.97	0.00033	0.98	0.94	0.00027	0.94
	GA2	DT	0.8	0.00037	0.88	0.74	0.00134	0.89	0.86	0.00041	0.90	0.94	0.00026	0.98
		BP	0.94	0.00024	0.94	0.95	0.00030	0.96	0.95	0.00044	0.95	0.94	0.00026	0.96
		ELM	0.95	0.00019	0.97	0.95	0.00043	0.96	0.97	0.00013	0.98	0.94	0.00060	0.96
	GA3	DT	0.86	0.00029	0.93	0.61	0.00192	0.91	0.88	0.00097	0.89	0.8	0.00060	0.97
		BP	0.94	0.00041	0.95	0.83	0.00067	0.93	0.96	0.00020	0.99	0.89	0.00052	0.91
		ELM	0.95	0.00063	0.97	0.91	0.00086	0.96	0.92	0.00032	0.96	0.86	0.00131	0.93

#### 3.4.3 Variable dimension reduction

The first five principal components for analysis were retained in this study. Only the DT model in the SWIR band improved the predictions of polysaccharides and ergosterol in the fruiting body powder (Table 5), increasing *R* ^2^ from 0.43 to 0.28 to 0.63 and 0.70, respectively. The predictive results from the other models were inferior to those of the original model, the ELM model achieved MSEP of 3.02734 and 2.49455 for polysaccharide prediction of GLP in SWIR and VNIR bands, respectively, probably because PCA is generally more effective for linear data; however, most hyperspectral data exhibit high-order correlations, affecting dimensionality reduction outcomes.

**TABLE 5:** Modeling and prediction results based on variable dimensionality reduction methods.

		SWIR						VNIR					
		GLC			GLP			GLC			GLP		
		R_P_^2^	MSEP	R_T_^2^	R_P_^2^	MSEP	R_T_^2^	R_P_^2^	MSEP	R_T_^2^	R_P_^2^	MSEP	R_T_^2^
polysaccharides	DT	0.50	0.49257	0.80	0.63	0.12218	0.72	0.54	0.17276	0.72	0.71	0.17911	0.85
	BP	0.32	0.38350	0.57	0.55	0.14353	0.81	0.57	0.25815	0.64	0.34	0.37401	0.43
	ELM	0.60	0.45253	0.83	0.53	3.02734	0.91	0.69	0.43786	0.95	0.38	2.49455	0.95
ergosterol	DT	0.62	0.00132	0.82	0.70	0.00114	0.74	0.68	0.00101	0.73	0.86	0.00039	0.93
	BP	0.48	0.00183	0.56	0.57	0.00096	0.66	0.58	0.00121	0.80	0.76	0.00087	0.76
	ELM	0.61	0.06103	0.89	0.53	0.01555	0.95	0.65	0.00445	0.92	0.74	0.00282	0.96

### 3.5 Visualization of polysaccharide and ergosterol content

**FIGURE 9:**
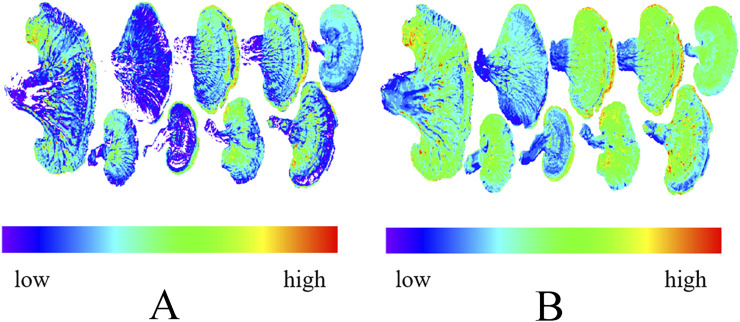
PCA diagram of *Ganoderma lucidum*. **(A)** Polysaccharides and **(B)** ergosterol.

We collected a complete hyperspectral image of *Ganoderma lucidum* with a stalk (Figure 9) and visualized the distribution of polysaccharide and ergosterol content. The results showed that the polysaccharide content of the *Ganoderma lucidum* stalk was significantly lower than that of the *Ganoderma lucidum* cap. The average levels of polysaccharide and ergosterol decreased following pulverization, which may explain why predictive accuracy for GLP was lower than that of the GLC. Additionally, pulverization improves sample homogeneity, increasing the spectral reflectance and potentially reducing prediction accuracy.

The whole *Ganoderma lucidum* fruiting body maintains its natural morphology and tissue structure, which may play a role in the synthesis, storage, and release of its active components. The structural integrity allows for interactions among various compounds, creating a relatively stable system that protects active components from environmental factors, such as oxidation. In contrast, pulverizing *Ganoderma lucidum* disrupts this natural structure, increasing its surface area and making it more susceptible to external factors, causing the degradation of active components and affecting predictive accuracy.

## 4 Discussion

HSI was used in this study to predict the chemical composition of the GLP and GLC. The results showed that the average predictive ability of the GLC was better than that of GLP. HSI technology was used to determine calcium and other trace elements in wheat grains and flour (Zhang et al., 2010). The results showed that the prediction accuracy of wheat grains was higher than that of wheat flour. This is consistent with the findings of the present study, in which intact samples yielded better predictions.

In the original BPNN and ELM models, polysaccharides and ergosterols achieved high prediction accuracies, *R* ^2^ > 0.9. However, in the GLP range of the SWIR band, the DT model showed a lower prediction accuracy, *R* ^2^ < 0.43, particularly ergosterol, with an *R* ^2^ of only 0.28. Furthermore, the predictive performance across models in the SWIR band is inferior to that in the VNIR band,and the MSEPs of the three models are significantly higher in the SWIR band. This may be attributed to the visible light and part of the near-infrared spectrum included in the VNIR band, providing extensive information on the color, texture, and chemical composition of objects. The SWIR band, while valuable, does not provide as abundant spectral information as that in the VNIR band. Common environmental substances, such as soil (Li et al., 2023a), show strong spectral responses in the VNIR band, and chlorophyll exhibits pronounced absorption features in the VNIR band (Sun et al., 2022). Additionally, the VNIR reflectance spectrum is less affected by water (Yang et al., 2023). VNIR band sensor technology is well-developed, facilitating data acquisition and processing with high spatial and spectral resolution. This technology has been extensively validated and applied in agriculture, forestry, geology, and environmental monitoring. In contrast, data from the SWIR band tends to be more complex, often resulting in large datasets that require more sophisticated algorithms to eliminate noise and manage other processing challenges. Therefore, advanced techniques are typically needed to handle SWIR data effectively. In summary, the VNIR band provides a better predictive performance than the SWIR band owing to its richer spectral content, higher data quality, and simpler processing methods, making it suitable for several applications.

In this study, characteristic wavelengths extracted through different GA methods were used to construct the predictive model. In most cases, models based on the characteristic wavelength performed better than those of the original model. The GA effectively extracts feature wavelengths, reducing noise and minimizing redundant input data, aligning with findings from other studies. Additionally, GA voting offers distinct advantages over GA iteration, probably owing to the inherent randomness in GA iteration processes, such as the initial population generation, crossover, and mutation, which may lead to inconsistent results. Each run can yield significantly different results, and the algorithm may become trapped in local optimal solution. Owing to its sequential nature, where each iteration depends on the previous results, greatly affected by the final results. In contrast, GA voting aggregates multiple independent GA results, favoring solutions that perform well across numerous runs, thus reducing the influence of randomness., This approach enhances the reliability and stability of the results, increasing the likelihood of finding a global optimal solution. The voting approach can efficiently screen for superior solutions and may achieve improved outcomes without requiring extensive iterations.

PCA dimensionality reduction often underperforms relative to the original model, likely because of its limited ability to capture the inherent structure and complexity of nonlinear data (Baytas et al., 2016). Additionally, the high sensitivity of PCA leads to deviations in the principal components, highlighting the need for data preprocessing to mitigate these deviations. Moreover, PCA reduces data dimensions by projection, potentially leading to the loss of some information. Furthermore, PCA assumes independence among input features. However, when features are highly correlated, PCA may fail to capture all data variances, negatively affecting the performance of the reduced-dimensional model.

This study examines how different feature wavelength extraction and regression algorithms perform in analyzing the chemical composition of Ganoderma lucidum in various states. The results indicate that the model combining feature wavelength extraction with machine learning algorithms effectively predicts the chemical components of Ganoderma lucidum. Among them, the ELM model built by GLC after voting feature selection was the most effective for polysaccharides and ergosterol prediction table. This study highlights the effectiveness of hyperspectral imaging in the SWIR and VNIR ranges for quickly evaluating quality parameters in Ganoderma lucidum and has potential applications in other traditional Chinese medicines. These findings offer important insights that could advance innovative approaches in this field and serve as a reference method for quality control in traditional Chinese medicines.

The application of HSI technology in traditional Chinese medicine (TCM) is well-documented and widely studied, but studies focused on *Ganoderma lucidum* remain limited. Despite the achievements made in this study, some limitations persist, such as a small sample size, variability in origin and strain, and the initial stages of model parameter optimization. Especially the small sample size of only 13 samples in the test set presents numerous challenges for model evaluation, generalization performance assessment, and data mining analysis. Therefore, future research should consider expanding the sample size to improve the accuracy and reliability of the results. Studies should also examine *Ganoderma lucidum* from diverse production areas and varieties, incorporate predictions for additional chemical components, and further refine model parameters.

HSI is a powerful analytical technology with broad application prospects and development potential. Simultaneously, the integration of artificial intelligence and big data expands the application of HSI technology, especially in fields of TCM, such as authenticity identification, quality evaluation, and origin traceability. It can also support monitoring of cultivation, harvest, and processing of TCM. HSI contributes to quality control and production process optimization, making outstanding contributions to standardizing TCMs.

## Data Availability

Data will be made available by the authors on request.

## References

[B1] Alfaro-Mejía E. Manian V. Ortiz J. D. Tokars R. P. (2023). A blind convolutional deep autoencoder for spectral unmixing of hyperspectral images over waterbodies. Front. Earth Sci. 11, 1229704. 10.3389/feart.2023.1229704

[B2] Baytas I. M. Lin K. Wang F. Jain A. K. Zhou J. (2016). Stochastic convex sparse principal component analysis. EURASIP J. Bioinform Syst. Biol. 2016, 15. 10.1186/s13637-016-0045-x 27660635 PMC5018037

[B3] Butt M. H. F. Ayaz H. Ahmad M. Li J. P. Kuleev R. (2022). A fast and compact hybrid CNN for hyperspectral imaging-based bloodstain classification. Congr. Evol. Comput. 2022, 1–8. 10.1109/CEC55065.2022.9870277

[B4] Chen L. T. Liu Z. X. Lan Y. Ma X. Wang R. J. (2024). Research on rice variety identification based on hyperspectral technology and principal component analysis. J. Agric. Sci. Technol. 10.13304/j.nykjdb.2023.0640

[B5] Chen Y. Y. Wang Z. B. (2019). Cross components calibration transfer of NIR spectroscopy model through PCA and weighted ELM-based TrAdaBoost algorithm. Chemom. Intelligent Laboratory Syst. 192, 192103824–103824. 10.1016/j.chemolab.2019.103824

[B6] Cör A. D. Knez Ž. Knez M. M. (2022). Antioxidant, antibacterial, antitumor, antifungal, antiviral, anti-inflammatory, and nevro-protective activity of Ganoderma lucidum: an overview. Front. Pharmacol. 13, 934982. 10.3389/FPHAR.2022.934982 35935849 PMC9353308

[B7] Cui X. Song Q. J. Zhang Y. Y. Xu G. Meng B. P. (2017). Estimation of soil organic carbon content in alpine grassland using hyperspectral data. Acta Prataculturae Sin. 10, 23–32.

[B8] Dai C. Sun J. Huang X. Zhang X. Tian X. Wang W. (2023). Application of hyperspectral imaging as a nondestructive technology for identifying tomato maturity and quantitatively predicting lycopene content. Foods 12 (15), 2957. 10.3390/FOODS12152957 37569225 PMC10418690

[B9] Dai J. Lu J. Lin R. C. Liu W. Y. (2002). Determination of nucleosides in siweilingzhi mixture by HPCE. China J. Chin. Materia Medica 09, 28–31. 12776566

[B10] Delwiche S. R. Rodriguez T. I. Rausch S. R. Graybosch R. A. (2019). Estimating percentages of fusarium-damaged kernels in hard wheat by near-infrared hyperspectral imaging. J. Cereal Sci. 87, 18–24. 10.1016/j.jcs.2019.02.008

[B11] Feng H. Chen Y. Song J. Lu B. Shu C. Qiao J. (2024). Maturity classification of rapeseed using hyperspectral image combined with machine learning. Plant Phenomics 06, 0139. 10.34133/PLANTPHENOMICS.0139 PMC10976948 38550661

[B12] He Z. Zhou S. (2022). BPNN-based behavioral modeling of the S-parameter variation characteristics of PAs with frequency at different temperatures. Micromachines (Basel) 13 (11), 1831. 10.3390/MI13111831 36363852 PMC9693541

[B13] Hou S. Shi H. Cao X. Zhang X. Jiao L. (2021). Hyperspectral imagery classification based on contrastive learning. IEEE Trans. Geoscience Remote Sens. 2021 (60), 1–13. 10.1109/TGRS.2021.3139099

[B14] Huang D. L. Chen X. K. Xu Y. Q. (2015). Analysis of different parts of ganoderma tsugae by fourier transform infrared spectroscopy. J. Anal. Sci. 31 (01), 59–62. 10.13526/j.issn.1006-6144.2015.01.013

[B15] Huang G. B. Zhu Q. Y. Siew C. K. (2006). Extreme learning machine: theory and applications. Neurocomputing 70 (01), 489–501. 10.1016/j.neucom.2005.12.126

[B16] Huang W. K. Huang Q. W. Guo Z. X. Zhang W. T. (2017). Determination on ganoderic acid A in ganoderma lucidum with different growth stages and different parts by HPLC. Edible Fungi China. 01, 60–62. 10.13629/j.cnki.53-1054.2017.01.013

[B17] Jia Z. Z. Du M. H. Wang P. X. (2024). Tool condition monitoring based on extreme learning machine and genetic algorithm. Nat. Sci. Available at: http://kns.cnki.net/kcms/detail/53.1223.N.20240424.1556.002.html.

[B18] Jiang W. Zhou G. S. Chen X. P. Ye H. Qiao Z. Bai G. G. (2018). Content determination of polysaccharides, triterpenoids and sterols of ganoderma lucidum pieces by ultraviolet-visible spectrophotometry. China Pharm. 14, 15–18.

[B19] Li M. Y. (2024a). Research on multi-factor stock selection model based on decision tree. Prod. Res. 02, 145–149. 10.19374/j.cnki.14-1145/f.2024.02.023

[B20] Li P. X. (2024b). Research on construction cost prediction of construction Project based on extreme iearning machine. Total Corros. Control (08), 92–94. 10.13726/j.cnki.11-2706/tq.2024.08.084.03

[B21] Li X. Li Z. Qiu H. Chen G. Fan P. (2023a). Soil carbon content prediction using multi-source data feature fusion of deep learning based on spectral and hyperspectral images. Chemosphere 336, 139161. 10.1016/J.CHEMOSPHERE.2023.139161 37302502

[B22] Li X. Peng F. Wei Z. Han G. Liu J. (2023b). Non-destructive detection of protein content in mulberry leaves by using hyperspectral imaging. Front. Plant Sci. 14, 1275004. 10.3389/FPLS.2023.1275004 37900759 PMC10602742

[B23] Li X. Wei Z. Peng F. Liu J. Han G. (2023c). Non-destructive prediction and visualization of anthocyanin content in mulberry fruits using hyperspectral imaging. Front. Plant Sci. 14, 1137198. 10.3389/FPLS.2023.1137198 37051079 PMC10083272

[B24] Liu H. Ma D. M. Liu X. K. Wang J. Y. (2016). Forecasting incidence of hand-foot-mouth disease with the ARIMA-BPNN combination model. Mod. Prev. Med. 16, 11–14+22.

[B25] Liu J. J. Huang W. H. Lv M. L. Si J. P. Guo B. L. Li S. J. (2011). Determination of ergosterol in ganoderma lucidum from different varieties and cultured tree species by HPLC. J. Chin. Med. Mater. 02, 30–33. 10.13863/j.issn1001-4454.2011.02.007 21823472

[B26] Liu T. Xu T. Y. Yu F. H. Yuan Q. Y. Guo Z. H. Xu Bo (2021a). A method combining ELM and PLSR (ELM-P) for estimating chlorophyll content in rice with feature bands extracted by an improved ant colony optimization algorithm. Comput. Electron. Agric. 186, 106177. 10.1016/J.COMPAG.2021.106177

[B27] Liu X. L. Liang G. Y. Yang Y. Wei F. X. Zhang Q. H. Deng T. F. (2023). Analysis of inorganic elements and nutrient components in different parts of Ganoderma Lucidum from Guizhou. J. Xinyang Normal Univ. Nat. Sci. Ed. 01, 45–51.

[B28] Liu Y. Long Y. Liu H. Lan Y. Long T. Kuang R. (2021b). Polysaccharide prediction in Ganoderma lucidum fruiting body by hyperspectral imaging. Food Chem. X 13, 100199. 10.1016/J.FOCHX.2021.100199 35498961 PMC9039882

[B29] Liu Y. Wang L. H. Yang L. B. Liu X. M. (2022). Drought prediction based on an improved VMD-OS-QR-ELM model. PLoS One 17 (01), e0262329. 10.1371/JOURNAL.PONE.0262329 34990468 PMC8735610

[B30] Lu C. Dong Y. N. Qiu X. H. (2024). An online study model based on ELM algorithm. Intelligent Comput. Applications 06, 116–124. 10.20169/j.issn.2095-2163.240615

[B31] Pan Z. C. Zhong Y. Fang L. Qi Z. C. Xu J. Liang Z. S. (2024). A rapid, hyperspectral-based method for determining sporoderm-broken rate of ganoderma lucidum spore powder. Chin. J. Mod. Appl. Pharm. (06), 38–44. 10.13748/j.cnki.issn1007-7693.20231852

[B32] Reddy P. Panozzo J. Guthridge K. M. Spangenberg G. C. Rochfort S. J. (2023). Single seed near-infrared hyperspectral imaging for classification of perennial ryegrass seed. Sensors (Basel) 23, 1820. 10.3390/S23041820 36850417 PMC9961513

[B33] Shi F. M. Tong X. R. Ding Z. M. Sun C. Liu W. S. (2013). Difference analysis of active components content in different medicinal parts of three species of ganoderma lucidum. China Med. Pharm. 21, 43–45+50.

[B34] Sun J. Yao K. Cheng J. Xu M. Zhou X. (2024). Nondestructive detection of saponin content in Panax notoginseng powder based on hyperspectral imaging. J. Pharm. Biomed. Anal. 242, 116015. 10.1016/J.JPBA.2024.116015 38364344

[B35] Sun W. Liu S. Zhang X. Zhu H. (2022). Performance of hyperspectral data in predicting and mapping zinc concentration in soil. Sci. Total Environ. 824, 153766. 10.1016/J.SCITOTENV.2022.153766 35151742

[B36] Wang H. Zhu H. Bi L. Xu W. Song N. Zhou Z. (2023). Quality grading of river crabs based on machine vision and GA-BPNN. Sensors (Basel) 23 (11), 5317. 10.3390/S23115317 37300045 PMC10255969

[B37] Wang M. Liu Y. P. Pei L. Li B. (2024a). Application of nuclear magnetic resonance hydrogen spectroscopy in quality control of ganoderma polysaccharides. Edible Fungi China. 02, 91–98. 10.13629/j.cnki.53-1054.2024.02.014

[B38] Wang T. T. Xu J. Liu X. L. (2024b). Research progress on ganoderma cultivation. J. Fungal Res. 01, 98–106. 10.13341/j.jfr.2023.1678

[B39] Wang Y. F. Zhang L. J. Duan H. P. (2021). Hydraulic performance optimization of radial diffuser multistage pump based on genetic algorithm-back propagation neural network. Sci. Technol. Eng. 04, 144–150.

[B40] Xia F. N. Guan X. Y. Chen S. D. Chen Q. Y. Zhang Y. F. Yang X. B. (2023). Fingerprint analysis and content determination of lipid components in ganoderma lucidum spore powder by HPLC-ELSD. Acta Edulis Fungi 06, 56–63. 10.16488/j.cnki.1005-9873.2023.06.006

[B41] Xiao Q. Bai X. Gao P. He Y. (2020). Application of convolutional neural network-based feature extraction and data fusion for geographical origin identification of radix astragali by visible/short-wave near-infrared and near infrared hyperspectral imaging. Sensors (Basel) 20 (17), 4940. 10.3390/s20174940 32882807 PMC7506783

[B42] Xu J. Xu D. Bai X. Yang R. Cao J. (2022). Non-destructive detection of moldy walnuts based on hyperspectral imaging technology. Molecules 27 (20), 6776. 10.3390/MOLECULES27206776 36296369 PMC9610789

[B43] Yang J. Xian L. Zhong T. S. Wei L. N. Li Y. R. Fu H. Y. (2017). Rapid identification of different kinds of Ganoderma lucidum based on near infrared spectroscopy fingerprint information. Lishizhen Med. Mater. Medica. Res. 28 (06), 1359–1361.

[B44] Yang Y. Nan R. Mi T. Song Y. Shi F. Liu X. (2023). Rapid and nondestructive evaluation of wheat chlorophyll under drought stress using hyperspectral imaging. Int. J. Mol. Sci. 24 (6), 5825. 10.3390/IJMS24065825 36982900 PMC10056805

[B45] Ye W. Yan T. Zhang C. Duan L. Chen W. Song H. (2022). Detection of pesticide residue level in grape using hyperspectral imaging with machine learning. Foods 11, 1609. 10.3390/FOODS11111609 35681359 PMC9180647

[B46] Yu Y. Sun D. L. (2024). Research on corrosion rate prediction of buried pipeline based on PCA-BPNN model. J. Lanzhou Univ. Technol. 04, 66–74.

[B47] Yuan S. Q. Shen Y. L. Zhang J. F. Yuan J. P. (2009). Performance predicting of centrifugal pumps with compound impeller based on improved BP neural network. Transactions of the Chinese Society for Agricultural Machinery 09, 31+83–86.

[B48] Zeng F. Q. Lu M. L. Xue Z. W. Peng N. (2023). Analysis of 18 main triterpenoids in basidiomata of Ganoderma lingzhi by HPLC. Mycosystema 11, 142–152. 10.13346/j.mycosystema.230095

[B49] Zhang M. Huang Y. K. Yuan Q. D. Zhao H. X. Huang L. Y. Guo D. L. (2024). Study on modeling and prediction of carbon paper base paper properties based on GA-BPNN algorithm. China Pulp and Pap. 43 (01), 121–127.

[B50] Zhang M. H. Zhang Y. Zhang Z. Q. Da H. Li L. Jia Y. C. (2024). Permafrost distribution innortheast China based on boosting regression tree. J. Harbin Inst. Technol. Available at: http://kns.cnki.net/kcms/detail/23.1235.T.20240624.1556.014.html.

[B51] Zhang Q. Q. (2020). Analytical study of triterpenoids from Ganoderma lingzhi fruiting bodies through infrared spectrocopy. University of Science and Technology of China. 10.27517/d.cnki.gzkju.2020.001539

[B52] Zhang Q. Q. Huang Q. (2021). Quantitative analysis of Ganoderma polysaccharides content in fruiting bodies by near-infrared spectroscopy. Mycosystema 01, 257–265. 10.13346/j.mycosystema.200218

[B53] Zhang S. Yin Y. Liu C. Li J. Sun X. Wu J. (2023). Discrimination of wheat flour grade based on PSO-SVM of hyperspectral technique. Spectrochim. Acta A Mol. Biomol. Spectrosc. 302, 123050. 10.1016/J.SAA.2023.123050 37379715

[B54] Zhang Y. Shi R. Rezaul K. M. Zhang F. Zou C. (2010). Iron and zinc concentrations in grain and flour of winter wheat as affected by foliar application. J. Agric. Food Chem. 58 (23), 12268–12274. 10.1021/jf103039k 21073194

